# Oxygen reserve index monitoring reduced the incidence of low pulse oxygen saturation during deep sedation for hysteroscopy: a prospective randomized controlled trial

**DOI:** 10.3389/fmed.2026.1732543

**Published:** 2026-02-18

**Authors:** Zheng Guan, Xin Li, Lin Liu, Jingjie Liu, Yanfeng Gao

**Affiliations:** 1 Department of Anesthesiology, The First Affiliated Hospital of Xi’an Jiaotong University, Xi’an, Shaanxi, China; 2 Department of Neurology, The Second Affiliated Hospital of Xi’an Jiaotong University, Xi’an, Shaanxi, China

**Keywords:** deep sedation, face mask, hysteroscopy, nasopharyngeal airway, oxygen reserve index, pulse oxygen saturation

## Abstract

**Objective:**

Low pulse oxygen saturation (SpO_2_) during deep sedation for hysteroscopy was common, which was a barrier for sedation performance. This randomized controlled trial tested whether oxygen reserve index (ORI) monitoring could reduce the incidence of low SpO_2_ during deep sedation for hysteroscopy.

**Methods:**

Based on whether ORI monitoring was adopted or not, and the oxygen supply methods during procedure [through face mask (FM) or nasopharyngeal airway (NPA)], four hundred participants underwent hysteroscopy under deep sedation were randomly divided into ORI+FM group, ORI+NPA group, non-ORI+FM group, and non-ORI+NPA group in a 1:1:1:1 ratio. Assist ventilation was performed when ORI dropped to zero in ORI monitoring groups, it was performed when SpO_2_ dropped to 98% in non-ORI monitoring groups. The incidence of low SpO_2_, which defined as SpO_2_ less than 95% during procedure, was compared.

**Results:**

Compared to non-ORI monitoring groups, the incidence of low SpO_2_ was lower (18% (95%CI: 0.103–0.257) and 21.4% (95%CI: 0.132–0.297) vs. 70.4% (95%CI: 0.612–0.796) and 51.5% (95%CI: 0.415–0.615), *P* < 0.001), average duration of assist ventilation (18 [0–35] and 15 [0–33] s vs. 36 [16–55] and 27 [0–53] s, *P* < 0.001) and time-weighted average duration of assist ventilation (0.016 [0–0.021] and 0.008 [0–0.016] vs. 0.022 [0.011–0.027] and 0.015 [0–0.025], *P* < 0.001) were shorter, the lowest SpO_2_ value during procedure was higher (98 [96–99] and 97 [96–99] vs. 93 [91–97] and 94 [92–99], *P* < 0.001) in ORI monitoring groups (data ordered as ORI+FM, ORI+NPA, non-ORI+FM, and non-ORI+NPA group). The absolute risk reduction between ORI monitoring groups and non-ORI monitoring groups was 0.41 (41%) (95%CI: 0.322–0.498), the NTT was 3. Compared to FM, NPA could induce mild to moderate sore throat and mild airway injury.

**Conclusion:**

During deep sedation for hysteroscopy, ORI monitoring and the altered clinician behavior from it may related to the reducing of the incidence of low SpO_2_ and ensure oxygenation in low-to-moderate risk outpatients.

**Clinical trial registration:**

https://clinicaltrials.gov/search?term=NCT05701839, identifier NCT05701839.

## 1 Introduction

Hysteroscopy was performed to diagnose and treat uterine diseases. Because of cervical dilatation and uterine distention during hysteroscopy, approximate 40% of patients referred considerable pain during anesthesia-free hysteroscopy (1), about 68.4% of patients referred moderate to severe pain immediately after hysteroscopy (2). Pain experience during hysteroscopy had negative impact on patient satisfaction (3). Sedation could relieve pain and improve patients’ comfort (4), therefore it was the most commonly used anesthesia method for hysteroscopy in Chinese mainland (5). Compared to conscious sedation, patients and gynecologists were more satisfied with deep level of sedation (6). But as the level of sedation deepened, the patients’ potential ability to maintain airway patency weakened (7), which may induce airway obstruction and hypoxemia.

Detecting impending hypoxemia before pulse oxygen saturation (SpO_2_) declined and timely airway intervention could reduce the incidence of hypoxemia. There was a correlation between SpO_2_ and oxygen partial pressure (PaO_2_) up to a PaO_2_ of 100 mmHg. For PaO_2_ value of more than 100 mmHg, SpO_2_ has reached a maximum (100%) and will not change any further. However, mixed venous oxygen saturation (SvO_2_) will continue to increase and reach a plateau when PaO_2_ exceeds more than 200 mmHg. Oxygen reserve index (ORI) (Masimo Corp., Irvine, CA, USA) was a new oxygenation monitoring parameter measured continuously and non-invasively by a specific pulse oximeter device, it detected variation of SvO_2_ once PaO_2_ reaches 100 mmHg, the value was from 0 to 1, and positive correlated with PaO_2_ between 100 and 200 mmHg (8). It may serve as an early warning indicator of impending hypoxemia when the value began to decline.

There were studies using ORI monitoring during intraoperative sedation under regional anesthesia, sedation for upper gastrointestinal endoscopy, and sedation for endoscopic retrograde cholangiopancreatography (9–11). These studies compared the effect of different sedatives on ORI value. There were not studies investigating the relationship between ORI monitoring and the incidence of low SpO_2_ during sedation for hysteroscopy.

In this study, we conducted a prospective, randomized controlled trial to investigate whether ORI monitoring could reduce the incidence of low SpO_2_ during deep sedation for hysteroscopy. We hypothesized that ORI monitoring may related to the reducing of the incidence of low SpO_2_ during this procedure.

## 2 Materials and methods

### 2.1 Approval and trial registry

This prospective, randomized controlled trial was conducted at the First Affiliated Hospital of Xi’an Jiaotong University in China between February 2023 and July 2024.

The study protocol adhered to the Consolidated Standards of Reporting Trials (CONSORT) guidelines (12). The procedure was conducted in accordance with the Helsinki Declaration-2013.^1^ The study was approved by the ethics committee of the First Affiliated Hospital of Xi’an Jiaotong University, Xi’an, Shaanxi, China (approval number: XJTU1AF2022LSK-402) on 25 October 2022, and was registered at the Clinicaltrials.gov (NCT05701839) on 28 December 2022 before the first participant was enrolled. Written informed consents were obtained from all the participants.

There were slight changes from the primary protocol. The threshold of ORI to perform assist ventilation was changed from 0.1 to zero in ORI monitoring groups. Because the SpO_2_ was usually 100% when ORI was 0.1, there may have excessive intervention in primary protocol. The threshold of SpO_2_ to perform assist ventilation was changed from 95 to 98% in non-ORI monitoring groups. Because the primary outcome was the incidence of SpO₂ less than 95%, it seems that there has been no attempt to reduce the primary outcome in primary protocol. The changes were occurred on January 2023 before the first participant was enrolled and decided by all authors. There was not accumulated outcome data before the protocol amendment occurred.

### 2.2 Participants characteristics and randomization

Participants scheduled for hysteroscopy under deep sedation were assessed for eligibility. Participants complied with all the following criteria could be enrolled in this study: aged between 18 and 60 years, the predicted procedure duration was more than 10 min. Participants met any of the following criteria could not be enrolled: body mass index (BMI) more than 30 kg/m^2^, predicted difficult airway, comorbidities including upper respiratory tract infection, pneumonia, obstructive sleep apnea syndrome, chronic obstructive pulmonary disease, asthma, coagulopathy, ongoing anticoagulant therapy, nasal deformity, and pregnant.

Based on whether ORI monitoring was adopted or not, and the oxygen supply methods during procedure [through face mask (FM) or nasopharyngeal airway (NPA)], participants were randomly divided into ORI+FM group, ORI+NPA group, non-ORI+FM group, and non-ORI+NPA group in a 1:1:1:1 ratio using random number table generated by Excel software. The specific method of random grouping was as follows: entered a set of data from 1 to 400 in column A, entered the formula “=RAND()” in column B1, and dropped down formula from B1 to B400 to generate random data, arranging the data in column B in ascending order. Participants were numbered serially according to the order of admission, the number above the line A100 (include A100) were divided into the ORI+FM group, the number between A101-A200 were divided into ORI+NPA group, the number between A201-A300 were divided into non-ORI+FM group, and the number below the line A301 (include A301) were divided into non-ORI+NPA group (The randomization list was provided as a Supplementary Table S3). The randomization codes were kept in sealed envelopes, they were opaque, tamper-proof and sequentially numbered. They were prepared and maintained by clinical trial assistant in our department who was not involved in this study. Once participants entered the operation room, the envelopes were given in order and opened by anesthesiologists. Participants were not informed of which oxygen supply methods and monitoring methods to use, the NPA was inserted after sedation. Outcome assessors were independent to anesthesiologists, they just assessed the severity of sore neck, sore jaw, and sore throat, they also calculated the time-weighted average duration of assist ventilation and performed the statistical analysis. Participants and outcome assessors were all blinded to group assignment.

### 2.3 Sedation strategies and airway intervention

Participants fasted solid and liquid for 8 and 2 h respectively, electrocardiogram, SpO_2_, blood pressure were monitored, the blood pressure measured every 3 min. ORI was monitored in ORI monitoring groups by finger-cot sensor which was worn on the participant’s right index finger, it was connected to ORI monitor (Masimo corp., Irvine, CA, USA). In non-ORI monitoring groups, the finger-cot sensor was worn, but the ORI was not monitored. 5 μg of sufentanil was injected firstly, followed by target-control infusion of propofol at a plasma concentration of 2–3 μg/mL, additional sufentanil was administered at the discretion of anesthesiologist. Sedative degree was evaluated using Ramsay Sedation Scale, the score should equal or more than five, namely participants had a sluggish response or no response to shaking or loud sound (13).

All the participants inhaled oxygen through FM at a flow rate of 5 L/min spontaneously for 5 min before sedation. In ORI+FM and non-ORI+FM groups, oxygen was supplied through FM continuously after sedation. In ORI+NPA and non-ORI+NPA groups, the NPA was inserted after sedation and oxygen was supplied through it. The oxygen flow rate was all of 5 L/min. Airway intervention includes chin lift, jaw thrust, neck extension, and assist ventilation. In ORI monitoring groups, assist ventilation through FM was performed when ORI dropped to zero, it lasted for another 10 s after ORI restored to 0.1. In non-ORI monitoring groups, assist ventilation through FM was performed when SpO_2_ dropped to 98%, it lasted for another 10 s after SpO_2_ restored to 100%. If assist ventilation was ineffective, laryngeal mask airway (LMA) would be inserted and mechanical ventilation would be performed, the data would be analyzed as the full analysis set. If systolic blood pressure was more than 180 mmHg, 20 μg of nitroglycerin would be used. If systolic blood pressure was less than 90 mmHg, 6 mg of ephedrine would be used.

### 2.4 Outcome measurement

The primary outcome was the incidence of low SpO_2_, which defined as SpO_2_ less than 95% during procedure. The secondary outcomes include: (1) Average duration of assist ventilation. (2) Time-weighted average duration of assist ventilation, which defined as the average duration of assist ventilation divided by procedure duration. (3) The severity of sore neck, sore jaw, and sore throat. The severity was evaluated using Visual Analogue Scale (VAS), 0 indicated no discomfort, 1–3 indicated mild discomfort, 4–6 indicated moderate discomfort, 7–10 indicated severe discomfort. (4) The airway injury, which defined as bleed or bloody secretion found on the tip of NPA or in the participants’ mouth or nose.

### 2.5 Sample size calculation and statistical analysis

The primary outcome was the incidence of low SpO_2_. Previous study showed that the incidence of SpO_2_ less than 95% during deep sedation for hysteroscopy was 51.2% (14), our pilot study showed that the incidence of SpO_2_ less than 95% with ORI monitoring was 34.4% (11/32). Using these data to calculate the sample size to achieve a statistical power of 0.9 and alpha error of 0.05 using two-sided test, accounting for 10% dropouts, both the ORI monitoring groups and the non-ORI monitoring groups required 200 participants.

The statistical analysis was performed using SPSS 26.0 software. We analyzed participants who were randomized as the full analysis set. The per protocol set, a subset of the full analysis set, included participants without major protocol deviations. The participants characteristics were analyzed according to the intention to-treat (ITT) principle. Due to major protocol deviations and the excessive missing proportion, the ITT analysis could not be performed for other data.

Data were assessed for normality using the Kolmogorov–Smirnov test. The normally distributed data were presented as mean ± SD, the non-normally distributed data were presented as median [interquartile range (IQR)], the categorical data were presented as number (percentage). Inter group comparison, the normally distributed data were compared using the Student *t*-test for independent samples, the non-normally distributed data were compared using the Kruskal-Wallis test, the categorical data were compared using the chi-square test. Intra group comparison using the repeated measures ANOVA. *p*-value of less than 0.05 was considered statistically significant. The Bonferroni correction was applied to adjust the statistical results in multiple comparisons.

For primary outcome, the absolute risk reduction (ARR) and number needed to treat (NNT) were also calculated. For secondary outcomes analysis, the comparisons of sore neck, sore jaw, sore throat, and airway injury were confirmatory, while the comparisons of incidence of SpO_2_ ≤ 90%, average duration of assist ventilation, time-weighted average duration of assist ventilation were exploratory.

## 3 Results

### 3.1 Participants characteristics data

**Figure 1:**
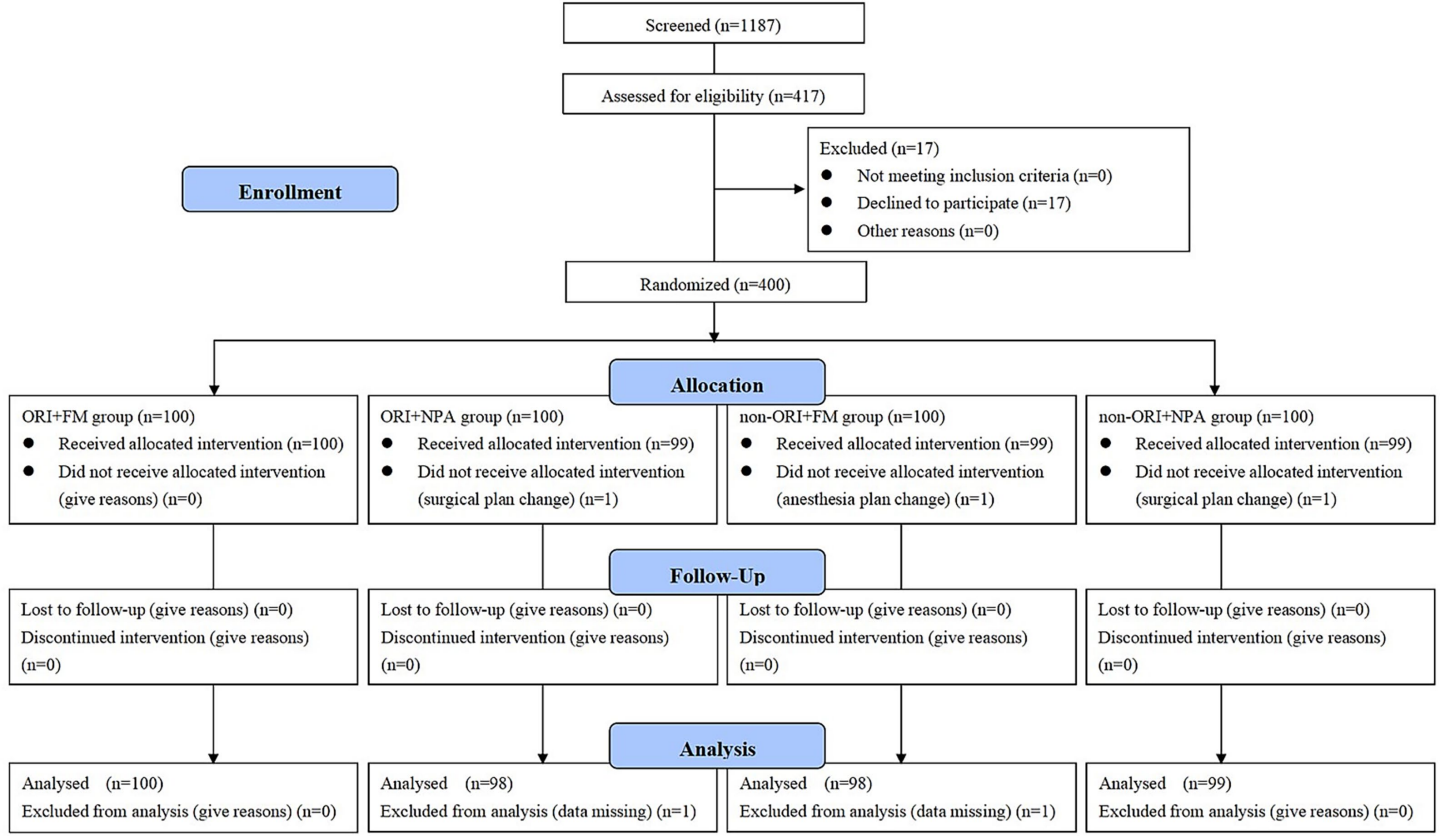
The CONSORT flow diagram. ORI, Oxygen reserve index; FM, Face mask; NPA, Nasopharyngeal airway.

Between February 2023 and July 2024, there were 1,187 participants scheduled for hysteroscopy in our hospital, in which 417 participants were eligible for this study, excluding 17 participants who declined to participate, finally 400 participants were enrolled and randomized in this study, 395 participants were completed the intervention and entered the statistical analysis as the per protocol set. The CONSORT flow diagram was showed in Figure 1. The details of the five excluded participants were described in Supplementary Table S1.

The demographic data were showed in Table 1. There were not significant differences of demographic data, comorbidities and preoperative hemoglobin between groups. Operational and anesthesia characteristics were showed in Table 2. there also were not significant differences of procedure types, procedure duration, anesthesia duration, and anesthetics dosage between groups.

**Table 1:** The demographic data between groups (full analysis set).

Parameters	ORI+FM group (*n* = 100)	ORI+NPA group (*n* = 100)	Non-ORI+FM group (*n* = 100)	Non-ORI+NPA group (*n* = 100)	Statistical value	*P-*value
Age (yrs)	40.0 ± 6.9	38.2 ± 6.0	39.2 ± 6.2	39.6 ± 7.6	1.338	0.262
BMI (kg/m^2^)	23 (21–24)	22 (21–24)	23 (21–24)	23 (21–25)	4.503	0.212
ASA physical status (*n*, %)					2.839	0.417
I grade	66 (66%)	69 (69%)	58 (58%)	65 (65%)		
II grade	34 (34%)	31 (31%)	42 (42%)	35 (35%)		
Smoking (*n*, %)	9 (9%)	9 (9%)	12 (12%)	8 (8%)	1.047	0.790
Hypertension (*n*, %)	31 (31%)	26 (26%)	36 (36%)	32 (32%)	2.362	0.501
Diabetes mellitus (*n*, %)	21 (21%)	16 (16%)	24 (24%)	18 (18%)	2.319	0.509
History of vaginal delivery (*n*, %)	53 (53%)	49 (49%)	58 (58%)	60 (60%)	2.990	0.393
History of hysteroscopy (n, %)	27 (27%)	27 (27%)	29 (29%)	34 (34%)	1.583	0.663
Menopause (n, %)	15 (15%)	17 (17%)	12 (12%)	10 (10%)	2.483	0.478
Hemoglobin (g/dl)	11.1 ± 1.5	11.5 ± 1.5	11.2 ± 1.3	11.5 ± 1.4	2.004	0.113

Categorical data were presented as number (percentage), continuous data were presented as mean ± SD for normally distributed data and median [interquartile range (IQR)] for non-normally distributed data. ORI, Oxygen reserve index; FM, Face mask; NPA, Nasopharyngeal airway, BMI: Body mass index; ASA, America Society of Anesthesiology.

**Table 2:** The operational and anesthesia characteristics between groups (per protocol set).

Parameters	ORI+FM group (*n* = 100)	ORI+NPA group (*n* = 98)	Non-ORI+FM group (*n* = 98)	Non-ORI+NPA group (*n* = 99)	Statistical value	*P-*value
Types of procedure (*n*, %)					6.905	0.864
Diagnose/biopsy	31 (31%)	36 (36.7%)	33 (33.7%)	35 (35.4%)		
Synechiolysis	18 (18%)	16 (16.3%)	15 (15.3%)	13 (13.1%)		
Polypectomy	20 (20%)	22 (22.4%)	21 (21.4%)	19 (19.2%)		
Myomectomy	19 (19%)	19 (19.4%)	23 (23.5%)	20 (20.2%)		
Combined procedure	12 (12%)	5 (5.1%)	6 (6.1%)	12 (12.1%)		
Procedure duration (min)	26 (18–33)	27 (17.8–35)	26 (18–33)	25 (18–31.3)	1.489	0.685
Anesthesia duration (min)	32 (25–40)	32 (22.8–40)	35 (25–41)	30 (24–36.3)	4.358	0.225
Anesthetics						
Sufentanil (μg)	10 (5–15)	10 (5–15)	10 (5–15)	10 (5–15)	4.807	0.187
Propofol (mg)	180 (150–242.5)	180 (150–220)	200 (170–240)	180 (150–220)	4.616	0.202
Propofol (mg/kg/min)	0.13 (0.12–0.15)	0.12 (0.13–0.15)	0.14 (0.13–0.15)	0.13 (0.13–0.15)	3.274	0.351

Categorical data were presented as number (percentage), continuous data were presented as median [interquartile range (IQR)]. ORI: Oxygen reserve index, FM: Face mask, NPA: Nasopharyngeal airway.

### 3.2 The vital signs between groups

**Figure 2:**
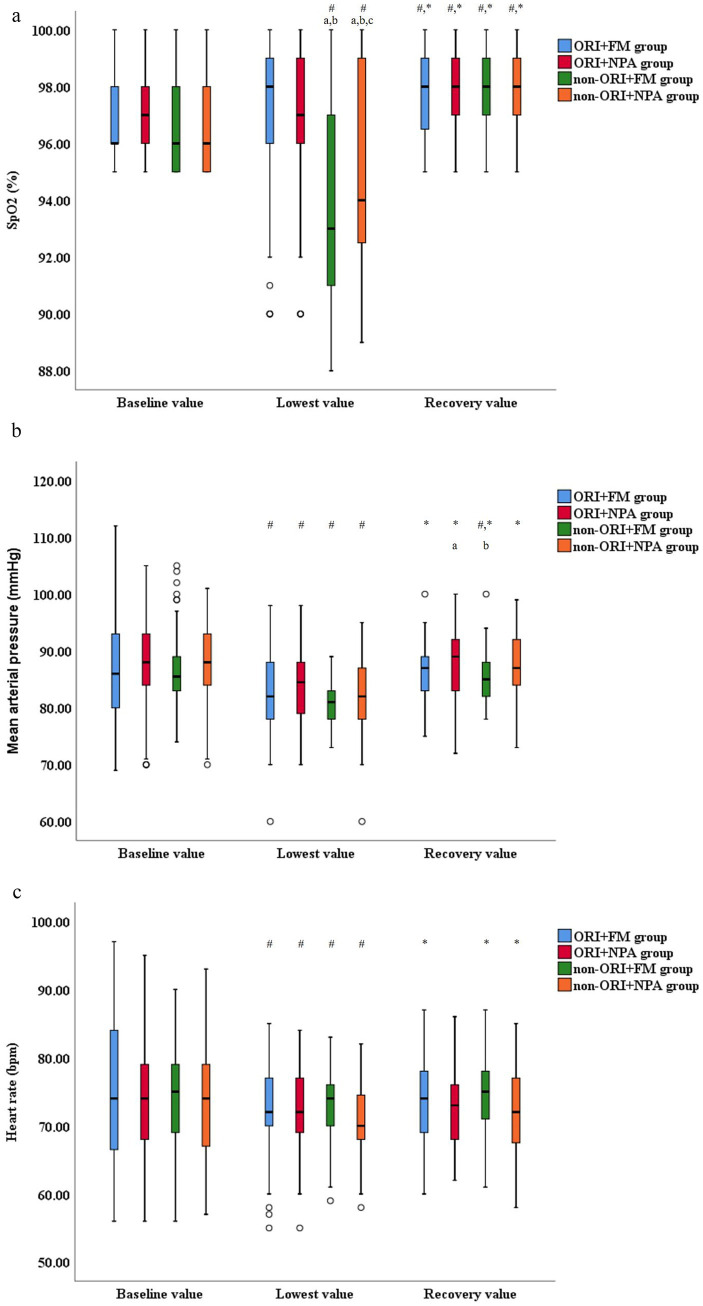
**(a)** The SpO_2_ fluctuations during anesthesia. SpO_2_, Pulse oxygen saturation; ORI, Oxygen reserve index; FM, Face mask; NPA, Nasopharyngeal airway. ^#^ Compare to baseline value, ^*^ Compare to lowest value, *P* < 0.05. ^a^ Compare to ORI+FM group, ^b^ Compare to ORI+NPA group, ^c^ Compare to non-ORI+FM group, *P* < 0.05. **(b)** The mean arterial pressure fluctuations during anesthesia. ORI, Oxygen reserve index; FM, Face mask; NPA, Nasopharyngeal airway. ^#^ Compare to baseline value, ^*^ Compare to lowest value, *P* < 0.05. ^a^ Compare to ORI+FM group, ^b^ Compare to ORI+NPA group, *P* < 0.05. **(c)** The heart rate fluctuations during anesthesia. ORI, Oxygen reserve index; FM, Face mask; NPA, Nasopharyngeal airway, bpm, Beat per minute. ^#^ Compare to baseline value, ^*^ Compare to lowest value, *P* < 0.05.

The vital signs during anesthesia were showed in Figures 2a–c and Supplementary Table S2. The SpO2, mean arterial pressure (MAP), and heart rate (HR) were recorded in three time points: 5 min after participants lied flat in operation room (baseline value), the lowest value during procedure (lowest value), 5 min after participants woke up (recovery value). The baseline and recovery values of SpO_2_ were measured during oxygen inhalation through FM at a flow rate of 5 L/min. Intra group comparison, the recovery value of SpO_2_ was higher than baseline and lowest values in all the groups, the baseline value of SpO_2_ was also higher than lowest value in non-ORI monitoring groups. The baseline and recovery values of MAP and HR were higher than lowest value in all the groups. Inter group comparison, the lowest value of SpO_2_ was higher in ORI monitoring groups than in non-ORI monitoring groups. The lowest value of SpO_2_ was also higher in non-ORI+NPA group than in non-ORI+FM group. There were not significant differences of baseline and recovery value of SpO_2_, all values of MAP and HR between groups.

### 3.3 The primary and secondary outcomes between groups

The primary and secondary outcomes were showed in Table 3. The incidence of low SpO_2_ was lowest in ORI monitoring groups, followed by non-ORI+NPA group, it was highest in non-ORI+FM group, there was not significant difference between ORI+FM group and ORI+NPA group. The ARR between ORI monitoring groups and non-ORI monitoring groups was 0.41 (41%) (95%CI: 0.322–0.498), the NTT was 3. The incidence of SpO_2_ ≤ 90% was also compared, it was highest in non-ORI+FM group, there were not significant differences between the other three groups. The ARR between ORI monitoring groups and non-ORI monitoring groups was 0.13 (13%) (95%CI: 0.073–0.187), the NTT was 8. The average duration of assist ventilation was shorter in ORI monitoring groups than in non-ORI monitoring groups. Time-weighted average duration of assist ventilation was shortest in ORI+NPA group, followed by ORI+FM and non-ORI+NPA groups, it was longest in non-ORI+FM group. The number of ORI dropped to zero were 2 (1–3) in ORI+FM group and 1.5 (0–3) in ORI+NPA group, there was not significant difference between groups.

**Table 3:** The primary and secondary outcomes between groups (per protocol set).

Parameters	ORI+FM group (*n* = 100)	ORI+NPA group (*n* = 98)	Non-ORI+FM group (*n* = 98)	Non-ORI+NPA group (*n* = 99)	Statistical value	*P-*value
Incidence of low SpO_2_ (*n*, %, 95CI)	18 (18%) (0.103–0.257)	21 (21.4%) (0.132–0.297)	69 (70.4%) (0.612–0.796) ^a^^,^^b^	51 (51.5%) (0.415–0.615) ^a^^,^^b^^,^^c^	77.305	< 0.001
Incidence of SpO_2_ ≤ 90% (*n*, %, 95CI)	3 (3%) (0.004–0.064)	3 (3.1%) (0.004–0.065)	24 (24.5%) (0.158–0.332) ^a^^,^^b^	8 (8.1%) (0.026–0.135) ^c^	35.080	< 0.001
The number of ORI dropped to zero	2 (1–3)	1.5 (0–3)	–	–	1.960	0.05
ADAV (seconds)	18 (0–35)	15 (0–33)	36 (16–55) ^a^^,^^b^	27 (0–53) ^b^	28.075	< 0.001
TWADAV (%)	0.016 (0–0.021)	0.008 (0–0.016) ^a^	0.022 (0.011–0.027) ^a^^,^^b^	0.015 (0–0.025) ^b^^,^^c^	36.495	< 0.001
Sore neck (*n*, %)					5.870	0.438
Mild	51 (51%)	50 (51%)	60 (61.2%)	59 (59.6%)		
Moderate	23 (23%)	25 (25.5%)	23 (23.5%)	24 (24.2%)		
Sore jaw (*n*, %)					3.968	0.681
Mild	55 (55%)	52 (53.1%)	58 (59.2%)	59 (59.6)		
Moderate	25 (25%)	18 (18.4%)	19 (19.4%)	17 (17.2%)		
Sore throat (*n*, %)					45.092	< 0.001
Mild	29 (29%)	45 (45.9%)	23 (23.5%) ^b^	37 (37.4%)		
Moderate	8 (8%)	20 (20.4%)	6 (6.1%) ^b^	25 (25.3%) ^a^^,^^c^		
Airway injury (*n*, %)	–	6 (6.1%)	–	5 (5.1%)	0.107	0.743

Categorical data were presented as number (percentage), continuous data were presented as median [interquartile range (IQR)]. ORI, Oxygen reserve index; FM, Face mask; NPA, Nasopharyngeal airway; SpO_2_, Pulse oxygen saturation; CI, Confidence interval; ADAV, Average duration of assist ventilation; TWADAV, Time-weighted average duration of assist ventilation.

a Compare to ORI+FM group.

b Compare to ORI+NPA group.

c Compare to non-ORI+FM group, *p* < 0.05.

There were not severe cardiovascular events, as well as aspiration, LMA insertion, mechanical ventilation, and unplanned admission in our study. The sore throat was more serious in ORI+NPA and non-ORI+NPA groups than in ORI+FM and non-ORI+FM groups. There were not significant differences of sore neck and sore jaw between groups. NPA insertion could induce mild airway injury, the incidence was 5.6% (11/197).

### 3.4 The subgroup analysis for primary outcome

To explore which population could benefit more from ORI monitoring, we performed subgroup analysis of primary outcome according to baseline characters, age ≤ 40 years or > 40 years, with diabetes or not, baseline SpO_2_ ≤ 95% or > 95%, procedure duration ≤ 25 min or > 25 min. The results were showed in Table 4. Compare to ORI monitoring groups, the incidences of low SpO_2_ were higher in non-ORI+FM group in all subgroup analysis, they were higher in non-ORI+NPA group in age ≤ 40 years, without diabetes, baseline SpO_2_ ≤ 95%, and all procedure duration subgroups. ARR and NNT analysis showed that patients under the age of 40, and with longer procedure duration were benefit more from ORI monitoring.

**Table 4:** The subgroup analysis of primary outcome: incidence of low SpO_2_ (per protocol set).

Parameters (n, %, 95CI)	ORI+FM group (*n* = 100)	ORI+NPA group (*n* = 98)	Non-ORI+FM group (*n* = 98)	Non-ORI+NPA group (*n* = 99)	ARR/NNT	Statistical value	*P-*value
Age							
≤ 40 years	8/52 (15.4%) (0.052–0.255)	10/58(17.2%) (0.072–0.273)	42/56(75%) (0.633–0.867) ^a^^,^^b^	34/56(60.7%) (0.475–0.739) ^a^^,^^b^	0.515/2	62.653	< 0.001
>40 years	10/48 (20.8%) (0.089–0.328)	11/40(27.5%) (0.198–0.352)	27/42(64.3%) (0.492–0.794) ^a^^,^^b^	17/43(39.5%) (0.243–0.548)	0.279/4	20.313	< 0.001
Diabetes							
With	4/21(19%) (0.007–0.374)	3/16(18.8%)(0.027–0.402)	15/24(62.5%) (0.416–0.834) ^a^^,^^b^	8/18(44.4%) (0.190–0.699)	0.358/3	12.153	0.007
Without	14/79(17.7%) (0.091–0.263)	18/82(22%) (0.128–0.311)	54/74(73%) (0.626–0.833) ^a^^,^^b^	43/81(53.1%) (0.420–0.642) ^a^^,^^b^	0.429/3	66.245	< 0.001
Baseline SpO_2_							
≤ 95%	9/51(17.6%) (0.068–0.285)	12/50(24%) (0.117–0.363)	38/49(77.6%) (0.654–0.897) ^a^^,^^b^	32/54(59.3%) (0.457–0.728) ^a^^,^^b^	0.471/3	49.809	< 0.001
>95%	9/49(18.4%) (0.071–0.296)	9/48(18.8%) (0.073–0.302)	31/49(63.3%)(0.493–0.773) ^a^^,^^b^	19/45(42.2%) (0.272–0.572)	0.346/3	29.509	< 0.001
Procedure duration							
≤ 25 min	0/48	1/43(2.3%) (−0.024–0.070)	21/45(46.7%) (0.315–0.618) ^a^^,^^b^	13/54(24.1%) (0.123–0.359) ^a^^,^^b^	0.332/4	43.290	< 0.001
>25 min	18/52(34.6%) (0.212–0.480)	20/55(36.4%) (0.232–0.495)	48/53(90.6%) (0.824–0.987) ^a^^,^^b^	38/45(84.4%) (0.734–0.955) ^a^^,^^b^	0.529/2	58.825	< 0.001

Data were presented as number (percentage). SpO_2_, Pulse oxygen saturation; CI, Confidence interval; ORI, Oxygen reserve index; FM, Face mask; NPA, Nasopharyngeal airway; ARR, Absolute risk reduction, NNT, Number needed to treat,

a Compare to ORI+FM group.

b Compare to ORI+NPA group, *P* < 0.05.

## 4 Discussion

Though the National Health Commission of the People’s Republic of China had focused on anesthesia and analgesia outside the operation room, a 2022 national survey conducted by Pain Group of Chinese Society of Anesthesiology showed that the overall sedation rate was just 67% for hysteroscopy. Patients’ safety concern, such as hypoxemia during sedation, was a barrier for the performance of sedation (15).

Respiratory complications were common during deep sedation for hysteroscopy. Studies showed that the incidence of upper airway obstruction was 2.9% (16), respiratory depression was 30–44.1% (17, 18), and SpO_2_ less than 95% was 51.2% (14). The risk factors of low SpO_2_ during deep sedation include high BMI and obesity (19, 20), long duration of operative procedures (21) and high dose of propofol (22). In our study, the incidence of SpO_2_ less than 95% was 60.9% in non-ORI monitoring groups. The incidence was higher than previous studies, the reason may be that we only enrolled participants with longer procedure duration.

SpO_2_ could conveniently monitor oxygenation status, but it did not decrease significantly until PaO_2_ less than 80 mmHg, so it has a delay in detecting low SpO_2_ (23). ORI reflected the state of moderate hyperoxia. The value began to decline 30 s earlier than SpO_2_ started to decline during anesthesia induction (24) and 200 s earlier than SpO_2_ started to decline during one-lung ventilation (25). During endotracheal intubation in intensive care unit patients, ORI fall below 0.4 preceded SpO_2_ fall below to 97%, the median time between these two events was 81 s (26). During general anesthesia intubation, the average time from ORI declined to zero to SpO_2_ declined to 94% was longer than the average time from SpO_2_ declined from 97 to 94%, the added warning time allowed for preventive treatment of impending hypoxemia (27). In our study, assist ventilation was performed when ORI declined to zero in ORI monitoring groups, while assist ventilation was performed when SpO_2_ declined to 98% in non-ORI monitoring groups. When ORI decreased to zero, SpO_2_ often remained at 99–100%, airway intervention was earlier in ORI monitoring groups than in non-ORI monitoring groups, so the incidence of low SpO_2_ was lower in patients with ORI monitoring than in patients without ORI monitoring, the lowest SpO_2_ was also higher in ORI monitoring groups than in non-ORI monitoring groups.

Besides, previous study showed that ORI value had positive correlation with PaO_2_ in the 100–200 mmHg in open heart surgery (28). 0.27 of the ORI value could distinguish PaO_2_ of more than 150 mmHg during one lung ventilation (29). ORI also can be used as a parameter to reflect preoxygenation efficiency during general anesthesia induction (30). In our study, we maintained ORI value greater than 0.1 in ORI monitoring groups, it could achieve mildly hyperoxia and reflect higher oxygen reserve. So average duration of assist ventilation and time-weighted average duration of assist ventilation were shorter in ORI monitoring groups.

ORI monitoring has been adopted during sedation. One study for intraoperative sedation under regional anesthesia showed that factors associated with a decrease in the intraoperative ORI to < 50% were diabetes mellitus and low baseline SpO_2_ (9). In our study, the baseline characters were consistent between groups. During sedation for upper gastrointestinal endoscopy using propofol, the incidence of ORI decreasing to zero was 65.7% (10), the incidence was 70.6% during sedation for endoscopic retrograde cholangiopancreatography (11). There has significant value using ORI monitoring during sedation, maintaining ORI above zero can effectively reduce the incidence of hypoxemia.

FM and NPA were the most commonly used devices for oxygen supply during sedation. NPA could alleviate upper airway obstruction by separating the soft palate from the throat (31), it has better oxygen supply effect. One study showed that the incidence of SpO_2_ less than 95% was 33% in FM oxygenation and 27% in NPA oxygenation during hysteroscopy (32). During sedation for gastrointestinal endoscopy for obese outpatients, comparing to nasal oxygen tube, NPA resulted in less incidence of hypoxemia (33), the respiratory depression was reduced from 13.5 to 1.9% (34). In our study, the incidence of low SpO_2_ was lower in non-ORI+NPA group than in non-ORI+FM group, the time-weighted average duration of assist ventilation was shorter in ORI+NPA and non-ORI+NPA groups than that in ORI+FM and non-ORI+FM groups. Our study also confirmed that NPA could better maintain upper airway opening than FM.

The airway intervention maneuvers, such as chin lift, jaw thrust, neck extension, and assist ventilation were often performed to alleviate respiratory depression and airway obstruction during sedation, they may related to sore neck and sore jaw (35). Because the assist ventilation duration was short in all the groups, so there were not significant differences of sore neck and sore jaw between groups. Because NPA was inserted into the nostril, the sore throat was more serious in ORI+NPA and non-ORI+NPA groups than in ORI+FM and non-ORI+FM groups. Another disadvantage of NPA insertion was the risk of nasopharyngeal injury. Studies showed that the incidence of minor nasopharyngeal injury was 4.7–26.1% (34, 36). Our study showed that the incidence of mild airway injury due to NPA insertion was 5.6%, and there was not moderate to severe airway injury.

Our study has several limitations. First, the intervention thresholds to perform assist ventilation were changed from the primary protocol, this may have an impact on study results. In addition, we did not update the protocol amendment on clinicaltrials.gov in time, which reducing the transparency of the protocol. Though we provided rigorous training to the anesthesiologists involved in the study, and tried our best to ensure their compliance with the protocol and implement interventions at the right time. The trial could not exclude operator behavior differences. Moreover, due to the lack of data on compliance to protocol, we were unable to provide an exact compliance rate to protocol. Second, pulmonary ventilation and gas exchange function has impact on SpO_2_, due to most patients were undergone outpatient hysteroscopy, pulmonary function examination was not performed in most of the patients and did not compare between groups. Third, NPA should be selected according to height and weight, we only used one model of NPA (ID = 6.0 mm) in our study, which may have an impact on ventilation effectiveness and the incidence of low SpO_2_. Finally, as an important evaluation indicator for sedation, patient-centered outcomes, such as patient satisfaction, recall of the procedure, and willingness to undergo a similar procedure in the future, were not collected, which may affect the self-reported VAS score for sore neck, sore jaw, and sore throat.

## 5 Conclusion

This study found that ORI monitoring may related to the reducing of the incidence of low SpO_2_ and ensure oxygenation during deep sedation for hysteroscopy. Because participants met criteria of high risk of hypoxemia were excluded in our study, the conclusion could only be generalized to low-to-moderate risk outpatients and could not be generalized to all populations. Further research is needed to investigate the benefits of ORI monitoring on high-risk outpatients. In addition, because anesthesiologists were not blinded to allocation, the observed benefit may reflect altered clinician behavior from ORI monitoring rather than intrinsic superiority of ORI monitoring.

## Data Availability

The original contributions presented in the study are included in the article/Supplementary material, further inquiries can be directed to the corresponding author.

## Footnotes

1
https://www.wma.net/policies-post/wma-declaration-of-helsinki/
