# Supplementary material for: Oxygen reserve index monitoring reduced the incidence of low pulse oxygen saturation during deep sedation for hysteroscopy: a prospective randomized controlled trial
Source: Front Med (Lausanne). 2026 Feb 18;13:1732543. doi: 10.3389/fmed.2026.1732543 (PMC12956626; doi:10.3389/fmed.2026.1732543)
Supplement: Supplementary file 1 [file Table_1.docx]

**Supplemental table S1 The demographic data of excluded participants.**

| Case number | Reason for exclusion | Location |
| --- | --- | --- |
| 1 | Surgical plan changed to hysterectomy | ORI+NPA group |
| 2 | The outcome data missed | ORI+NPA group |
| 3 | Anesthesia plan changed to spinal anesthesia | non-ORI+FM group |
| 4 | The anesthesia characteristics and outcome data missed | non-ORI+FM group |
| 5 | Surgical plan changed to laparoscopic surgery | non-ORI+NPA group |
