# Supplementary material for: Oxygen reserve index monitoring reduced the incidence of low pulse oxygen saturation during deep sedation for hysteroscopy: a prospective randomized controlled trial
Source: Front Med (Lausanne). 2026 Feb 18;13:1732543. doi: 10.3389/fmed.2026.1732543 (PMC12956626; doi:10.3389/fmed.2026.1732543)
Supplement: Supplementary file 2 [file Table_2.docx]

**Supplemental table S2 Variation in vital signs during anesthesia (per protocol set).**

| Parameters | ORI+FM group (*n*=100) | ORI+NPA group (*n*=98) | non-ORI+FM group (*n*=98) | non-ORI+NPA group (*n*=99) | Statistical value | *P-*value |
| --- | --- | --- | --- | --- | --- | --- |
| Baseline SpO_2_ (%) | 96 (96-98) | 97 (96-98) | 96 (95-98) | 96 (95-98) | 4.585 | 0.205 |
| Lowest SpO_2_ (%) | 98 (96-99) | 97 (96-99) | 93 (91-97) ^#,a,b^ | 94 (92-99) ^#,a,b,c^ | 64.486 | ˂ 0.001 |
| Recovered SpO_2_ (%) | 98 (96-99) ^#,*^ | 98 (97-99) ^#,*^ | 98 (97-99) ^#,*^ | 98 (97-99) ^#,*^ | 2.771 | 0.428 |
| Statistical value | 21.675 | 13.348 | 43.179 | 28.763 |  |  |
| *P-*value | ˂ 0.001 | 0.001 | ˂ 0.001 | ˂ 0.001 |  |  |
| Baseline MAP (mmHg) | 86 (80-93) | 88 (84-93) | 86 (83-89) | 88 (84-93) | 6.108 | 0.106 |
| Lowest MAP (mmHg) | 82 (78-88) ^#^ | 85 (79-88) ^#^ | 81 (78-83) ^#^ | 82 (78-87) ^#^ | 11.168 | 0.051 |
| Recovery MAP (mmHg) | 87 (83-89) ^*^ | 89 (83-92) ^*,a^ | 85 (82-88) ^#,*,b^ | 87 (84-92) ^*^ | 17.390 | 0.001 |
| Statistical value | 55.189 | 12.848 | 33.285 | 31.441 |  |  |
| *P-*value | ˂ 0.001 | 0.002 | ˂ 0.001 | ˂ 0.001 |  |  |
| Baseline HR (bpm) | 75.1±10.1 | 74.0±8.4 | 74.3±7.5 | 73.4±8.3 | 0.625 | 0.599 |
| Lowest HR (bpm) | 71.9±6.0 ^#^ | 72.2±5.6 ^#^ | 72.9±4.8 ^#^ | 70.9±4.8 ^#^ | 2.530 | 0.057 |
| Recovery HR (bpm) | 73.7±6.3 ^*^ | 73.0±5.6 | 74.5±5.1 ^*^ | 72.3±6.1 ^*^ | 2.625 | 0.050 |
| Statistical value | 36.336 | 22.632 | 30.918 | 11.212 |  |  |
| *P-*value | ˂ 0.001 | ˂ 0.001 | ˂ 0.001 | ˂ 0.001 |  |  |

Continuous data were presented as mean±SD for normally distributed data and median [interquartile range (IQR)] for non-normally distributed data. ORI: Oxygen reserve index, FM: Face mask, NPA: Nasopharyngeal airway, SpO_2_: Pulse oxygen saturation, MAP: Mean arterial pressure, HR: Heart rate, bpm: Beat per minute. ^#^ Compare to baseline value, ^*^ Compare to lowest value, *P* ˂ 0.05. ^a^ Compare to ORI+FM group, ^b^ Compare to ORI+NPA group, ^c^ Compare to non-ORI+FM group, *P* ˂ 0.05.
